# Formation of a Thin Continuous GaSb Film on Si(001) by Solid Phase Epitaxy

**DOI:** 10.3390/nano8120987

**Published:** 2018-11-28

**Authors:** Evgeniy Chusovitin, Sergey Dotsenko, Svetlana Chusovitina, Dmitry Goroshko, Anton Gutakovskii, Evgeniy Subbotin, Konstantin Galkin, Nikolay Galkin

**Affiliations:** 1 Institute of Automation and Control Processes FEB RAS, 5 Radio St., 690041 Vladivostok, Russia; niksard@yandex.ru (E.C.); docenko@mail.dvo.ru (S.D.); goroshko@iacp.dvo.ru (D.G.); jons712@mail.ru (E.S.); galkinkn@iacp.dvo.ru (K.G.); ngalk@iacp.dvo.ru (N.G.); 2 Far Eastern Federal University, School of Natural Sciences 8 Sukhanova St., 690950 Vladivostok, Russia; 3 Rzhanov Institute of Semiconductor Physics SB RAS, 13 Lavrentieva Ave., 630090 Novosibirsk, Russia; gut@isp.nsc.ru

**Keywords:** solid phase epitaxy, crystal structure, epitaxial relationships, GaSb, Si(001)

## Abstract

Nanocrystalline GaSb films were grown on Si(001) from the stoichiometric Ga–Sb mixture using solid-phase epitaxy at temperatures of 200–500 °C. Use of the solid-phase epitaxy method allowed the suppression of Ga surface diffusion and prevention of intense Sb desorption. At the annealing temperature of 300 °C, a 14-nm-thick GaSb film aggregates, while a 20-nm-thick GaSb film remains continuous with a roughness of 1.74 nm. A GaSb film with a thickness of 20 nm consists of crystalline grains with a size of 9–16 nm. They were compressed by ~2%. For some GaSb grains, new epitaxial relationships have been found: GaSb(111)||Si$\left(11\bar{1}\right)$ and GaSb$\left[11\bar{2}\right]$||Si$\left[1\bar{1}0\right]$, GaSb(113)||Si$\left(11\bar{1}\right)$ and GaSb$\left[1\bar{1}0\right]$||Si$\left[1\bar{1}0\right]$, and GaSb$\left(11\bar{1}\right)$||Si(002) and GaSb$\left[1\bar{1}0\right]$||Si$\left[1\bar{1}0\right]$.

## 1. Introduction

The integration of III–V semiconductor optoelectronic components with silicon technology, in particular those based on gallium antimonide (GaSb), is currently an important task for the semiconductor industry and fundamental science [1]. Gallium antimonide heteroepitaxy on a clean silicon substrate would be the simplest way for large-scale integration. However, this approach proved difficult due to a number of reasons: a large lattice mismatch (~12%) between GaSb and silicon; a large difference between their thermal expansion coefficients (about 3 times) [2]; and a difference in the chemical bonds of the crystal lattice—GaSb is an ion crystal, while Si is a covalent one. These issues usually result in a high density of dislocations propagated through the entire film [3] and in the appearance of antiphase boundaries [4]. The defects considerably reduce the performance of the GaSb/Si heterostructure.

At present, many studies are underway to find the optimal conditions for the formation of a defect-free GaSb film on Si(001) (temperature regime, substrate miscut angle, buffer layers, etc.) [5,6,7,8,9,10,11,12,13]. The most common way to grow a GaSb film on silicon is via molecular beam epitaxy (MBE). One of the main problems in the formation of a continuous GaSb film by MBE is a high surface diffusion of Ga atoms [5], which results in the formation of a low concentration of nucleation centers; as a result, large GaSb crystalline blocks with sizes up to 200 nm are formed [6]. In this case, relatively thin films (with a thickness of 20 nm) exhibit a significant roughness of 35 nm [5]. To suppress the surface diffusion of Ga atoms, either various buffer layers are used (AlSb epitaxial layer [5,7,8,9,12,13]; Si(001)2 × 2–Ga and Si(001)2 × 3–Ga surface reconstructions [10]; the thin SiO_2_ layer [11]), or the formation temperature of the GaSb film is significantly decreased (down to 200 °C) [6]. Lowering of the formation temperature allows not only the suppression of the Ga surface diffusion, but also reduction of Sb desorption during the MBE process. Provided a low growth temperature is used, one can reduce the Sb/Ga molecular flux ratio from 8.5–10 at a growth temperature of 560–600 °C [10,12] down to 5 at 200 °C [6]. To further reduce the surface diffusion of Ga atoms during GaSb film formation, solid-phase epitaxy (SPE) can be used instead of MBE. In the SPE process, the deposition of the Ga–Sb mixture occurs on an unheated substrate. In this case, since no desorption of Sb occurs [14] and Ga diffusion is strongly suppressed, Ga and Sb can be deposited in a 1:1 ratio.

The aim of our work was to form a continuous and smooth GaSb film on a clean Si(001) surface by the SPE method using a stoichiometric Ga–Sb mixture. According to electron energy loss spectra, GaSb formation takes place during annealing at 200 °C. It was shown that a 20-nm-thick GaSb film, after annealing at 300 °C, remains continuous and smooth, a root-mean square roughness (*σ*_rms_) is 1.74 nm. At the same time, a 14-nm-thick GaSb film does not withstand annealing at 300 °C and aggregates into connected islands.

## 2. Materials and Methods

For the formation of all the samples, a phosphorous-doped silicon substrate with surface orientation (001) and a resistivity of 7.5 Ω·cm was used. Gallium and antimony were deposited from Knudsen cells. All growth procedures were carried out in an Omicron ultra-high vacuum chamber (Omicron NanoTechnology GmbH, Taunusstein, Germany) with a base pressure of 2 × 10^−11^ Torr. The chamber was equipped with an Auger electron spectroscopy (AES) unit that could record the spectra of electron energy loss spectroscopy (EELS), and with a low-energy electron diffraction (LEED) unit. The substrate temperature was controlled by an infrared pyrometer. Gallium and Sb deposition rates (~0.34 nm/min) were calibrated using LEED patterns of known surface reconstructions of Ga and Sb on Si(001) [15,16]. Native oxide was removed from the substrate surface by annealing at 1160 °C for 20 min, and as a result, the surface reconstruction Si(001)2 × 1 was formed; contamination of the surface was below the detection limit of AES.

Formation of GaSb thin films by the SPE method was carried out in two steps: (i) a stoichiometric Ga–Sb mixture (14 or 20 nm) was grown by co-deposition of Ga (99.99%) and Sb (99.999%) on the unheated surface with Si(001)2 × 1 reconstruction; (ii) then, the mixture was annealed (Table 1). Sample *A* was annealed at 200–500 °C in increments of 50 °C for 15 min at each temperature, while Sample *B* was annealed only twice at 200 and 300 °C for 15 and 20 min, respectively. The GaSb film study consisted of two steps. In the first step (sample *A*), the temperature stability of the GaSb film was studied in the temperature range of 200–500 °C, and it was established that the maximum annealing temperature for the formation of a continuous GaSb film was 300 °C. In the second step (sample *B*), the deformation and structure of the continuous GaSb film were investigated. The substrate was not intentionally heated during the deposition of the Ga–Sb mixture; however, since the distance from the Ga and Sb sources to the substrate was about 7 cm, the substrate was gradually heated up to 150–170 °C by the end of the Ga–Sb mixture deposition. In situ control of GaSb formation was performed by monitoring the appearance and dynamics of GaSb-characteristic EELS peaks [17]. After unloading, the sample surface was studied by atomic force microscopy (AFM, NT-MDT Spectrum Instruments, Moscow, Russia), while a structure of the grown films was analyzed using high-resolution transmission electron microscopy (TEM, JEM-4000EX, JEOL Ltd., Tokyo, Japan) of the sample cross-section in the zone axis [110]. Epitaxial relationships (ERs) and the lattice parameters of the continuous GaSb film were determined by analyzing fast Fourier transform (FFT) patterns. Optical properties were investigated by a Bruker Vertex 80v spectrophotometer. Quantitative analysis of AFM images was carried out using Balagan’s Grain Analysis v.1.0 software [18].

## 3. Results and Discussion

**Figure 1:**
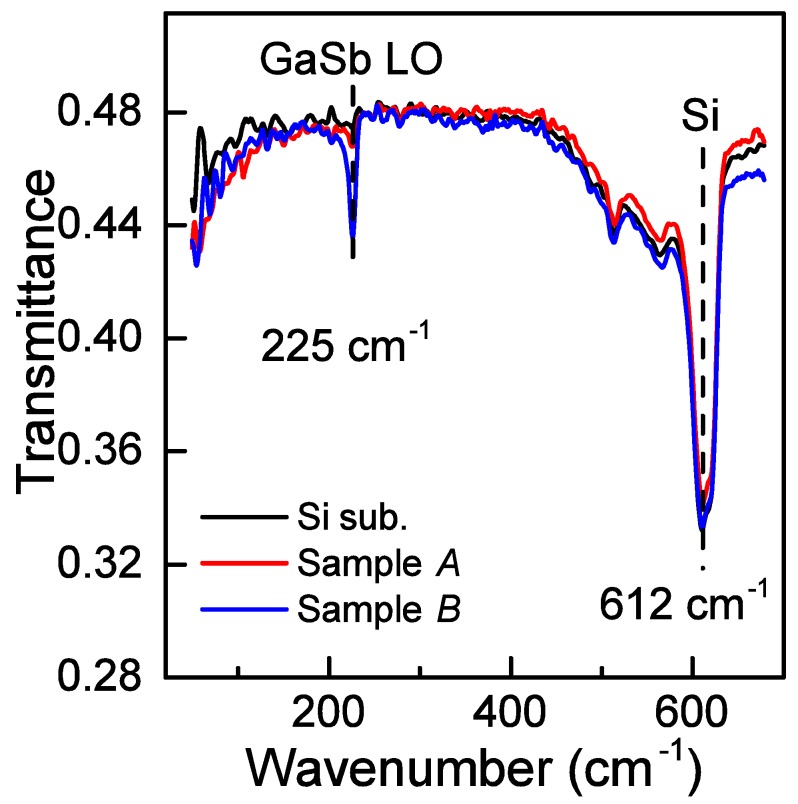
Spectral dependence of transmittance in the far infrared (FIR) region for the silicon substrate and samples *A* and *B*.

The formation of GaSb films by the SPE method was confirmed by the appearance of an absorption peak at 225 cm^−1^ on the spectra in the far infrared (FIR) spectral region (Figure 1). The observed peak corresponds to the longitudinal optical phonon (LO) [19] in the GaSb cubic lattice (F-43m). The peak at 612 cm^−1^ comes from the silicon substrate (Figure 1). One can see that a decrease in the thickness of the GaSb film from 20 down to 14 nm resulted in a significant decrease (about four-fold) in the intensity of the 225 cm^−1^ peak, which is due to the aggregation of the GaSb film on the sample *A* surface during the annealing at 300–500 °C (see the discussion of the AES, LEED, and AFM data obtained for sample *A*).

**Figure 2:**
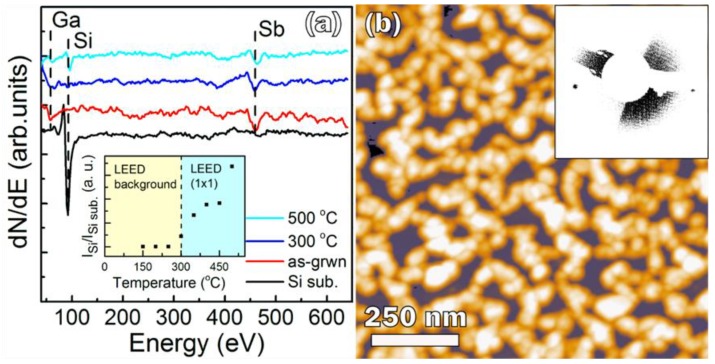
The evolution of the Auger spectra in the process of sample *A* formation; the inset is a dependence of the intensity of the silicon peak (92 eV) on the annealing temperature (the minimal temperature in the inset is the temperature of the Ga–Sb mixture after deposition) (**а**); atomic force microscopy (AFM) image of sample *A* after the final annealing at 500 °C for 15 min; the inset is a 1 × 1 LEED pattern that appears after annealing of the film at 300 °C for 15 min (**b**).

The as-deposited 14-nm-thick Ga–Sb stoichiometric mixture completely covers the substrate surface (sample *A*), which is confirmed by the disappearance of the 92-eV silicon peak in the Auger spectrum and by the appearance of Ga (55 eV) and Sb (454 eV) peaks (Figure 2a). In addition, the Si(001)2 × 1 LEED pattern completely disappeared, indicating a disordered surface. Since the Si peak did not appear after annealing at 200–250 °C, the film was continuous at these temperatures. After annealing of the mixture film at 300 °C, the Si peak appears again (Figure 2a, inset), as well as the Si(001)1 × 1 LEED pattern (Figure 2b, inset). At 300 °C, the diffusion of Si atoms from the substrate into the GaSb film is small. Therefore, the observed Si peak and the LEED pattern originate from the Si substrate surface, which was uncovered because of the GaSb film agglomeration. Since the silicon peak intensity is very low at 300 °C, there is no significant GaSb film aggregation. The rapid increase of the Si peak with the increase of the annealing temperature (Figure 2a, inset) resulted from intense GaSb film agglomeration, which is confirmed by the AFM data (Figure 2b). The AFM image shows that after the final annealing (500 °C), an array of connected GaSb islands is formed, while about 30% of the substrate surface is free from GaSb. The islands’ concentration is rather high, at 4.3 × 10^10^ cm^−2^; their average lateral size is 50 nm and height is 21.3 nm. Because of agglomeration, the 14-nm-thick GaSb film became very rough, with *σ*_rms_ = 7.05 nm. The discontinuity of the film is also confirmed by the FIR absorption spectroscopy data: a low intensity of the peak at 225 cm^−1^ was caused by the decrease in GaSb film surface coverage (Figure 1). Summarizing the results obtained for sample *A*, we can state that: (i) the 14-nm-thick GaSb film was continuous after annealing at 200–250 °C, (ii) the GaSb film agglomeration took place during annealing at 300–500 °C, (iii) there is no significant GaSb film aggregation at 300 °C, and (iv) the GaSb film thickness of 14 nm is not sufficient to withstand annealing at temperatures of 300 °C and higher.

**Figure 3:**
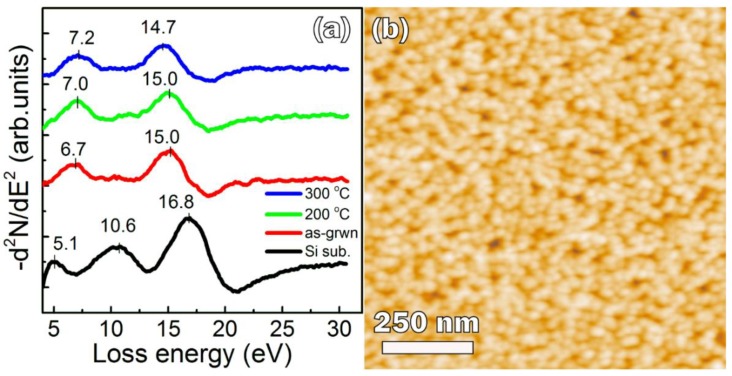
Evolution of the spectra of electron energy loss spectroscopy (EELS) spectra in the process of sample *B* formation (**a**); AFM image of sample *B* after the final annealing at 300 °C for 20 min (**b**).

Therefore, for sample *B*, we increased the film thickness by about 50%—up to 20 nm—and set the maximum annealing temperature at 300 °C because at this temperature, there is no significant film aggregation, but it is high enough for GaSb formation [6]. After deposition of a 20-nm-thick Ga–Sb mixture, only GaSb peaks in the EELS spectrum were seen: at 6.7 and 15.0 eV [17,20] (Figure 3a). The peak at 15.0 eV is closer to the bulk plasmon in crystalline cubic GaSb (14.7–14.8 eV [17] and 14.7 [20]) rather than in amorphous GaSb (14.3 eV [21]), so we can state that a crystalline GaSb film with a cubic F-43 m lattice was formed.

According to the theory of plasma oscillations [22], the value of the bulk plasmon energy $\mathrm{\hbar}\omega_{p}$ of GaSb is proportional to the square root of the valence electron concentration $n_{v}$ in GaSb:

(1) $$\mathrm{\hbar}\omega_{p}=A\;\sqrt{n_{v}}$$

where *A* is a constant which must be calculated for GaSb. To calculate this constant, we used the formula:

(2) $$A=\frac{\mathrm{\hbar}\omega_{p}^{sc}}{\sqrt{n_{v}^{sc}}},$$

where $\mathrm{\hbar}\omega_{p}^{sc}$ and $n_{v}^{sc}$, respectively, are the energy of a bulk plasmon and the concentration of valence electrons for a relaxed GaSb single crystal. The valence electron concentration in relaxed single-crystal GaSb is $n_{v}^{sc}$ = $n_{v}^{ideal}$ + *n* − *p*, where $n_{v}^{ideal}=N/a^{3}$ = 32/(0.609593 nm)^3^ = 1.41 × 10^23^ cm^−3^ is the valence electrons’ concentration in the ideal cubic GaSb, and *n* and *p* are the electron and hole concentrations in relaxed single-crystal GaSb, respectively. It was found that Czochralski-grown unintentionally *p*-doped relaxed single-crystal GaSb has hole concentration *p* ≈ 1.3 × 10^17^ cm^−3^ [23]. Therefore, we can assume that for this *p*-doped relaxed single-crystal GaSb, *n* << *p* << $n_{v}^{ideal}$, and hence $n_{v}^{sc}$ ≈ $n_{v}^{ideal}$ = 1.41 × 10^23^ cm^−3^, while the energy of the bulk plasmon of the GaSb single crystal $\mathrm{\hbar}\omega_{p}^{sc}$ = 14.7 eV [17,20]. We placed these values of $\mathrm{\hbar}\omega_{p}^{sc}$ and $n_{v}^{sc}$ in Equation (2) and obtained *A* = 3.91 × 10^‒11^ eV·cm^3/2^.

The high value of bulk plasmon energy of the as-grown GaSb film (e.g., 15 eV) in Figure 3a results from the higher valence electron concentration (1.47 × 10^23^ cm^−3^—the value is calculated by Equation (1)) compared to the ideal GaSb structure. The change in the valence electron concentration *n_v_*, and hence the shift energy of the bulk plasmon (e.g., 0.3 eV for plasmon energy of 15 eV), can originate from a change in the unit cell volume as a result of deformation.

Therefore, we consider the influence of GaSb lattice deformation Δ​a/a on the bulk plasmon energy $\mathrm{\hbar}\omega_{p}$. Substituting Equation (2) into Equation (1), we obtain the equation for the calculation of the bulk plasmon energy of the real GaSb film:

(3) $$\mathrm{\hbar}\omega_{p}=\frac{\mathrm{\hbar}\omega_{p}^{sc}}{\sqrt{n_{v}^{sc}}}\;\sqrt{n_{v}}$$

The concentration of valence electrons depends on the deformation of the lattice by the formula $n_{v}=\frac{N}{{\left(a+\Delta a\right)}^{3}}+n-p$, where Δ*a* is the change of the lattice constant of GaSb. The GaSb film grown in our conditions is *p*-type, thus for this film *n* << *p*, so we neglected contribution of electron concentration *n* in the formula. According to our Hall measurements, the hole concentration in a continuous GaSb film (sample *B*) is 1.6 × 10^18^ cm^−3^. It is higher than that in single crystal GaSb, but it is much less than the concentration of valence electrons of GaSb $n_{v}^{ideal}$ = 1.41 × 10^23^ cm^−3^. Therefore, the contribution of the hole concentration *p* to $n_{v}$ is very small, so we ignored it. The number of valence electrons, *N*, is obtained from the formula for an ideal crystal $n_{v}^{ideal}=N/a^{3}$; then

(4) $$n_{\nu }=n_{\nu }^{ideal}{\left(1+\frac{\Delta a}{a}\right)}^{-3}$$

Substituting Equation (4) into Equation (3) and assuming $n_{v}^{sc}$ ≈ $n_{v}^{ideal}$, we obtain the equation for the estimation of the influence of deformation, Δ*a*/*a*, on the bulk plasmon energy, $\mathrm{\hbar}\omega_{p}$:

(5) $$\mathrm{\hbar}\omega_{p}=\mathrm{\hbar}\omega_{p}^{sc}{\left(1+\Delta a/a\right)}^{-\frac{3}{2}}$$

By applying Equation (5), we have found that the bulk plasmon energy $\mathrm{\hbar}\omega_{p}$ = 15 eV resulted from GaSb lattice compression of 1.33%. If we took into account the contribution of the hole concentration *p* into the value of Δ*a*/*a*, then the obtained value would differ from that calculated from Equation (5) only by −3.6 × 10^−4^%. It is very small value, so we neglected it. According to our assessment, during the GaSb mixture deposition, the substrate was gradually heated by Ga and Sb sources up to 170 °C at the end of the deposition process. This temperature is sufficient for the crystallization of the GaSb mixture [24], therefore GaSb crystals appeared in the film during deposition. During the crystallization, noticeable compression of the GaSb lattice (1.33%) occurs. Further annealing at 200 °C did not change the bulk plasmon energy (Figure 3a), and so it did not change the value of GaSb lattice deformation. We suppose that raising the temperature by 30 °C (up to 200 °C) cannot lead to remarkable recrystallization and reduce the deformation of the GaSb lattice. An increase in the annealing temperature up to 300 °C results in a decrease of the bulk plasmon energy (14.7 eV). This value of bulk plasmon energy corresponds to the relaxed GaSb film. Thus, the annealing at a temperature of 300 °C reduces the deformation in the GaSb film that was induced in the film during its crystallization.

The EELS peak at 6.7 eV, which appeared on the EELS spectra just after the deposition of the Ga–Sb mixture, is probably a superposition of the peaks originating from interband transitions (5.1–5.3 eV) and transitions from filled surface states to dangling bond levels (7.5–7.6 eV) [17]. The presence of a single peak instead of two is most likely due to the insufficient resolution of our analyzer. In contrast to the bulk plasmon, with annealing, the 6.7 eV peak shifts to a higher energy (Figure 3a). Since the intensity of the interband transitions depends on the volume of the material [17], which does not change during the annealing, the shift of the 6.7 eV peak up to 7.2 eV should be assumed to be an increase of the contribution of transitions to dangling bond levels. This implies an increase of the contribution from the surface states, the number of which grows due to the increase of the GaSb film roughness during the annealing. Therefore, we assume that the observed high-energy shift of the EELS peak at 6.7 eV results from the increase of GaSb film roughness during the annealing.

The AFM image shows that the 20-nm-thick GaSb film covers the whole substrate (Figure 3b) and consists of nanocrystals with sizes of some tens of nanometers. The maximum hole depth between nanocrystals is less than 10 nm. It has a roughness of *σ*_rms_ = 1.74 nm (Table 1), which is much lower than that obtained for GaSb films grown by MBE on clean silicon (*σ*_rms_ ≈ 35 nm, at a film thickness of ≈20 nm) [5] and an AlSb buffer layer (*σ*_rms_ ≈ 5–6 nm) [5]. The surface relief development during GaSb MBE growth arises from a large Ga surface diffusion coefficient, while in the case of SPE, Ga, and Sb atoms intermixed enough to form small crystalline grains at the very beginning of the annealing, at about 200 °C. The increase of the annealing temperature results in grain size growth, but without noticeable development of the film roughness.

**Figure 4:**
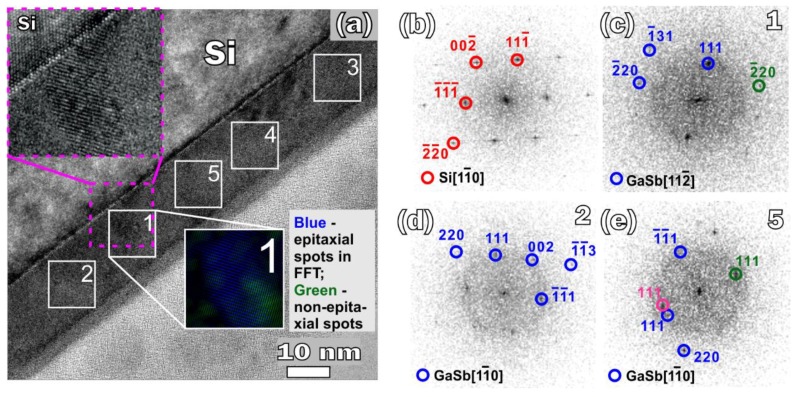
TEM images of the cross-section of sample *B*; squares 1–5 mark areas with individual GaSb grains, which have an interface with the substrate (**a**); in the insets one can see the fast Fourier transform (FFT) filtered image of area 1 and a magnified image of the interface between GaSb grain in area 1 and the substrate (magenta frame). The FFT pattern taken from the silicon substrate (**b**), and from areas 1, 2, and 5, respectively (**c**–**e**). Spots marked by green and magenta circles in FFT patterns are produced by GaSb grains not presented in Table 2.

According to the results of TEM, the SPE-grown GaSb film is polycrystalline, consisting of grains with sizes of 9–16 nm (Figure 4). The crystalline structure of the GaSb film was determined at several areas by analyzing the fast Fourier transform (FFT) patterns (Figure 4c–e; Table 2). The grains are characterized by both a compressive (up to −2.58%) and tensile stress (up to 0.9%) and they are disoriented relative to each other by an angle of 14–30°. To calculate the average deformation of GaSb film, the lattice deformation of 10 GaSb grains presented in the TEM image was determined. Among them five grains are not listed in Table 2 as they have no interface with the substrate, thus no epitaxial relationship can be deduced for these grains. The average deformation of the GaSb film is −0.03%. The deformation value is slightly different from zero because of limited number of analyzed grains in the TEM image compared with those probed by electron beam of EELS, but it corresponds to the fully relaxed film. The epitaxial relationships (ERs) of the GaSb grains with the substrate are very diverse; most of them have not been previously described in the literature. The most frequently encountered ERs were: GaSb(111)||Si$\left(11\bar{1}\right)$ and GaSb$\left[11\bar{2}\right]$||Si$\left[1\bar{1}0\right]$ (area 1), GaSb(113)||Si$\left(11\bar{1}\right)$ and GaSb$\left[1\bar{1}0\right]$||Si$\left[1\bar{1}0\right]$ (area 2), and GaSb$\left(11\bar{1}\right)$||Si(002) and GaSb$\left[1\bar{1}0\right]$||Si$\left[1\bar{1}0\right]$ (area 3) (Table 2, Figure 4a,c,d).

The ERs of GaSb(111)||Si(111) and GaSb$\left[1\bar{1}0\right]$||Si$\left[1\bar{1}0\right]$ (area 4) and GaSb(111)||Si(220) and GaSb$\left[1\bar{1}0\right]$||Si$\left[1\bar{1}0\right]$ (area 5) completely coincide with the relations obtained for GaSb grown by MBE [7,11]. Besides, the ER for area 5 is rarely observed. In our paper [25] devoted to GaSb nanocrystals SPE-grown on Si(001) and embedded in a silicon matrix, we obtained only one ER for all the nanocrystals: GaSb(111)||Si(111) and GaSb$\left[1\bar{1}0\right]$||Si$\left[1\bar{1}0\right]$. The ER looks differ from the ER obtained for GaSb nanocrystals formed by high-dose ion implantation followed by GaSb crystallization inside the silicon lattice: GaSb(002)||Si(002) and GaSb$\left[1\bar{1}0\right]$||Si$\left[1\bar{1}0\right]$ [26]. However, due to the symmetry of the Si and GaSb crystals, if one observes ER GaSb(111)||Si(111) and GaSb$\left[1\bar{1}0\right]$||Si$\left[1\bar{1}0\right]$, the following relationships for the matching planes should also be observed: GaSb(220)||Si(220) and GaSb(002)||Si(002). So, we can state that the ERs observed for GaSb nanocrystals grown both by SPE and by ion-beam synthesis are identical. This means that when GaSb crystallizes directly from a Si crystal lattice during MBE [7,11], ion-beam synthesis [26], or SPE of a thin layer of GaSb (less 5 nm) [25], the only ER is GaSb(111)||Si(111) and GaSb$\left[1\bar{1}0\right]$||Si$\left[1\bar{1}0\right]$. In this case, the matching direction relationship is GaSb$\left[1\bar{1}0\right]$||Si$\left[1\bar{1}0\right]$, which leads to a mismatch of 12.2% in this direction. On the contrary, when SPE films are formed from the Ga–Sb mixture, many different ERs are observed (Table 2). A variety of ERs indicates that GaSb crystallization begins at not only the Si/GaSb interface, but also all over the film bulk. A different characteristic of the film crystallization is explained by the fact that during the deposition of the Ga–Sb mixture, the substrate temperature gradually increased from room temperature up to ~170 °C, due to the heating of the substrate caused by the Ga and Sb sources. Since the diffusion of Ga atoms over the surface and in the bulk of the Ga–Sb mixture film is significantly hampered compared to its diffusion over crystalline Si or GaSb surfaces, and since a temperature of 170 °C is sufficient for the GaSb crystallization [24], crystal nucleation takes place all over the Ga–Sb film. If a GaSb grain has no interface with the substrate (isolated grain), it can be arbitrarily oriented relative to the substrate, as one can see in Figure 4a (FFT filtration inset, green grain) and Figure 4c. A disorientation angle calculated for planes GaSb(111) and GaSb$\left(\bar{2}20\right)$ of isolated grains – namely, angle between GaSb(111) and Si(111) planes, and angle between GaSb$\left(\bar{2}20\right)$ and Si(220) planes – was in the range of −14–26°; only in one case was the value about 0.1°. On the contrary, all the GaSb grains in areas 1–5 had a sharp interface with the substrate (Figure 4a, magenta frame inset) and epitaxial orientation (see disorientation angle in Table 2). Being most likely, their crystallization began from the substrate, while the new ERs could form under the influence of a GaSb_grain/Ga–Sb_mixture interface and the neighboring isolated grains, because when only the Si substrate influences crystallization, the only ERs that can be observed is GaSb(111)||Si(111) and GaSb$\left[1\bar{1}0\right]$||Si$\left[1\bar{1}0\right]$. Thus, during SPE growth of a GaSb film, crystallization begins both in the film bulk and at the film/substrate interface.

**Figure 5:**
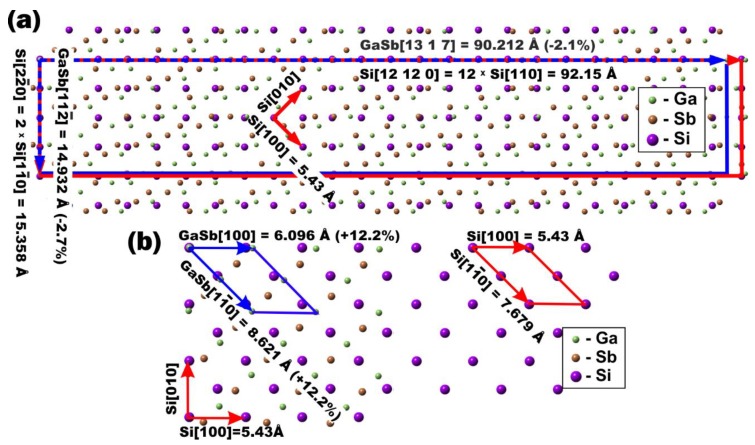
The arrangement of atoms on the GaSb/Si(001) interface for the following ERs: GaSb(111)||Si$\left(11\bar{1}\right)$ and GaSb$\left[11\bar{2}\right]$||Si$\left[1\bar{1}0\right]$ (**a**) and GaS(111)||Si(111) and GaSb$\left[1\bar{1}0\right]$||Si$\left[1\bar{1}0\right]$ (**b**). The lattice mismatch М is shown in parentheses after the length of the GaSb lattice vector.

To estimate the deformation of the GaSb unit cell on the Si(001) surface, we calculated the mismatch between the GaSb and Si lattices in selected directions on the Si(001) surface for the ERs corresponding to the areas 1 and 4 (Figure 5a,b). The lattice mismatch *M* for lattice vectors GaSb[*U*_1_*V*_1_*W*_1_] and Si[*UVW*] of 2D cells of GaSb and Si, was calculated by the following equation:

(6) $$M=\frac{\left(b_{\left[U_{1}V_{1}W_{1}\right]}^{GaSb}-b_{\left[UVW\right]}^{Si}\right)}{b_{\left[UVW\right]}^{Si}},$$

where $b_{\left[U_{1}V_{1}W_{1}\right]}^{GaSb}=a_{GaSb}\sqrt{U_{1}^{2}+V_{1}^{2}+W_{1}^{2}}$ is the length of vector [*U*_1_*V*_1_*W*_1_] of the 2D GaSb lattice; $b_{\left[UVW\right]}^{Si}=a_{Si}\sqrt{U^{2}+V^{2}+W^{2}}$ is the length of vector [*UVW*] of the 2D Si lattice; *a_GaSb_* and *a_Si_* are the lattice constants of GaSb and Si, respectively. The *b* values are shown in Figure 5a,b. The ER for area 4, GaSb(111)||Si(111) and GaSb$\left[1\bar{1}0\right]$||Si$\left[1\bar{1}0\right]$, is common for MBE-grown GaSb, while the ER for area 1, GaSb(111)||Si$\left(11\bar{1}\right)$ and GaSb$\left[11\bar{2}\right]$||Si$\left[1\bar{1}0\right]$, was observed only for SPE-grown GaSb; below, we will refer to this ER as *GS*$\left[11\bar{2}\right]$. It attracted our attention because of a small discrepancy between the GaSb and Si (−2.7%) lattices in the direction of GaSb$\left[11\bar{2}\right]$ (lattice vectors GaSb$\left[11\bar{2}\right]$ and Si$\left[2\bar{2}0\right]$ in Figure 5a).

While for GaSb(111)||Si(111) and GaSb$\left[1\bar{1}0\right]$||Si$\left[1\bar{1}0\right]$ in the direction of GaSb$\left[1\bar{1}0\right]$, the mismatch is 12.2% (Figure 5b), for *GS*$\left[11\bar{2}\right]$ ER on the Si(001) surface, together with the GaSb$\left[11\bar{2}\right]$||Si$\left[1\bar{1}0\right]$ epitaxial direction, there is another possible direction with a mismatch of −2.1%: GaSb[13 1 7]||Si[110] (lattice vectors GaSb[13 1 7] and Si[12 12 0] in Figure 5a). Although *GS*$\left[11\bar{2}\right]$ ER is incommensurate (Figure 5a), the difference in the mismatch along GaSb$\left[11\bar{2}\right]$ and GaSb[13 1 7] is only 0.6%. The maximum mismatch value of *GS*$\left[11\bar{2}\right]$ ER is about four times smaller than that for the GaSb[100]||Si[100] direction (12.2%), which coexists with GaSb$\left[1\bar{1}0\right]$||Si$\left[1\bar{1}0\right]$ on the Si(001) surface for the ER of GaSb(111)||Si(111) and GaSb$\left[1\bar{1}0\right]$||Si$\left[1\bar{1}0\right]$ (Figure 5b). The arrangement of atoms shown in Figure 5b is realized at MBE and leads to significant deformation of the GaSb crystal, which is relaxed by dislocations localized at the GaSb/Si(001) interface [25]. In the case of embedding into the Si matrix, the edge dislocations give rise to threading dislocations in the Si cap layer [25]. The new arrangement of atoms found for SPE-grown GaSb and shown in Figure 5a induced a smaller deformation in the GaSb crystal, and hence could cause a lower dislocation density at the GaSb/Si(001) interface. Thus, the *GS*$\left[11\bar{2}\right]$ ER is very suitable for GaSb embedding into the Si matrix. Determining the growth conditions that allow the formation of a GaSb film with the only ER being *GS*$\left[11\bar{2}\right]$ will be the task of our future work.

## 4. Conclusions

A continuous polycrystalline GaSb film on Si(001) was grown by SPE without the use of any buffer layers. New epitaxial relationships between GaSb and Si have been found: GaSb(111)||Si$\left(11\bar{1}\right)$ and GaSb$\left[11\bar{2}\right]$||Si$\left[1\bar{1}0\right]$ (*GS*$\left[11\bar{2}\right]$), GaSb(113)||Si$\left(11\bar{1}\right)$ and GaSb$\left[1\bar{1}0\right]$||Si$\left[1\bar{1}0\right]$, and GaSb$\left(11\bar{1}\right)$||Si(002) and GaSb$\left[1\bar{1}0\right]$||Si$\left[1\bar{1}0\right]$, which were not observed in the MBE-grown GaSb. The new ERs originate from GaSb grains for which crystallization begins inside at the Si_substrate/Ga-Sb_mixture interface under the influence of surrounding amorphous mixture and neighboring isolated grains. The most interesting ER is *GS*$\left[11\bar{2}\right]$, for which the smallest mismatch between GaSb and Si lattices was observed: −2.7% and −2.1% in the directions GaSb$\left[11\bar{2}\right]$ and GaSb[13 1 7], respectively. The mismatch values were at least four times lower than that for MBE-grown GaSb (12.2%). The conditions for the formation of a continuous GaSb film on Si(001) by SPE were determined: the Ga–Sb mixture film thickness should be not less than ~20 nm and the maximum annealing temperature should be about 300 °C. The obtained results show that SPE growth of GaSb can help to reduce the defectiveness of GaSb/Si heterostructures.

## Figures and Tables

**Table 1:** Sample formation conditions and morphological parameters of sample surface.

Sample	GaSb Mixture Thickness, nm	Time of Deposition, min	Annealing Mode	*σ*_rms_, nm	GaSb Islands		
					Concentration, ×10^10^ cm^−2^	Height, nm	Lateral Size, nm
*A*	14	20	200–500 °C (step 50 °C)— for 15 min	7.05	4.3	21.3	50
*B*	20	30	200 °C—15 min, 300 °C—20 min	1.74	–	–	–

**Table 2:** The epitaxial relationships observed for GaSb grains in the film of sample B that have an interface with silicon substrate.

Area	Epitaxial Relationships	Angle of Disorientation with Substrate	Deformation of the GaSb Lattice	MBE Method
1	GaSb(111)||Si$\left(11\bar{1}\right)$ ^a^ GaSb$\left[11\bar{2}\right]$||Si$\left[1\bar{1}0\right]$ ^a^	0	−0.61%	-
2	GaSb(113)||Si$\left(11\bar{1}\right)$ ^a^ GaSb$\left[1\bar{1}0\right]$||Si$\left[1\bar{1}0\right]$ ^a^	2.0	−0.38%	-
3	GaSb$\left(11\bar{1}\right)$||Si(002) ^a^ GaSb$\left[1\bar{1}0\right]$||Si$\left[1\bar{1}0\right]$ ^a^	1.8	−1.73%	-
4	GaSb(111)||Si(111) ^a^ GaSb$\left[1\bar{1}0\right]$||Si$\left[1\bar{1}0\right]$ ^a^	0	−2.58%	GaSb{002}||Si{002} ^b^ GaSb〈110〉||Si〈110〉 ^b^ GaSb(111)||Si(111) ^c^ GaSb$\left[1\bar{1}0\right]$||Si$\left[1\bar{1}0\right]$ ^c^
5	GaSb(220)||Si$\left(11\bar{1}\right)$ ^a^ GaSb(111)||Si(220) ^a^ GaSb$\left[1\bar{1}0\right]$||Si$\left[1\bar{1}0\right]$ ^a^	0	−2.00%	GaSb{111}||Si{220} ^b^ GaSb〈110〉||Si〈110〉 ^b^

^a^ This work. ^b^ Reference [7]. ^c^ Reference [11].
